# Isolation and characterization of plant growth promoting endophytic bacteria from the rhizome of *Zingiber officinale*

**DOI:** 10.1007/s13205-013-0143-3

**Published:** 2013-06-06

**Authors:** B. Jasim, Aswathy Agnes Joseph, C. Jimtha John, Jyothis Mathew, E. K. Radhakrishnan

**Affiliations:** School of Biosciences, Mahatma Gandhi University, Priyadharshini Hills PO, Kottayam Dist, Kerala, 686560 India

**Keywords:** Endophytic bacteria, Indole 3 acetic acid, HPLC, *Pseudomonas* sp., 16S rDNA sequencing

## Abstract

Endophytes, by residing within the specific chemical environment of host plants, form unique group of microorganisms. Microbially unexplored medicinal plants can have diverse and potential microbial association. The rhizome of ginger is very remarkable because of its metabolite richness, but the physiological processes in these tissues and the functional role of associated microorganisms remain totally unexplored. Through the current study, the presence of four different endophytic bacterial strains were identified from ginger rhizome. Among the various isolates, ZoB2 which is identified as *Pseudomonas* sp. was found to have the ability to produce IAA, ACC deaminase and siderophore. By considering these plant growth promoting properties, ZoB5 can expect to have considerable effect on the growth of ginger.

## Introduction

Plants are associated with a diverse community of microorganisms. The microorganisms residing within the plants or endophytes are unique in their adaptations to specific chemical environment of host plant. Even some of these microorganisms are shared genetically with the molecular machinery for the synthesis of plant specific compounds. This makes endophytes to be considered as untapped source of natural products (Rosenblueth and Martínez-Romero 2006; Strobel 2003). Endophytes also provide advantages to the host plant by producing plant growth regulators, by providing resistance to diseases and also by assisting in phytoremediation (Lodewyckx et al. 2002).

The mechanisms by which endophytes deal with ever-changing environmental conditions may provide better survival advantages to host plants. The evolution of endophytic biochemical pathways for the production of plant growth hormones is very interestingly present in plants (Strobel 2003). Endophytic microorganisms can vary based on the plant source, age, type of tissue, season of sampling, and environment. Generally, the concentration of the endophytic bacteria is more at the root than at shoot tissue (Zinniel et al. 2002). A large number of plant species are shown to be associated with bacteria like *Pseudomonas, Bacillus, Azospirillum* etc. (Chanway 1996). At the same time various species of bacteria can be associated with specific plants as in the case of rice where bacteria like *Pantoea, Azospirillum, Methylobacterium, Rhizhobium, Herbaspirillum, Burkholderia* etc. are found to be endophytically associated. These bacterial species have been shown to have added contribution to the yield and growth of the rice plants (Mano and Morisaki 2008). Hard wooded trees are also shown to be endophytically associated with bacteria such as *Serratia* sp., *Rahnella* sp., *Pseudomonas* sp., *Stenotrophomonas* sp. etc. (Taghavi et al. 2009). So by considering the remarkable features of ginger rhizome, much diverse and even specific bacterial association can be well expected.

Endophytic bacteria have been reported from wide variety of plants but the functional role is known only with limited number of isolates. One of the major contributions of these microorganisms towards plant growth is the production of auxin-like molecules (Spaepen et al. 2007). Indole 3 acetic acid (IAA) being an auxin can stimulate both rapid responses like cell elongation and long term responses like cell division and differentiation in plants (Taghavi et al. 2009). Indole 3 acetic acid (IAA) is shown to be produced by many root associated bacteria including *Enterobacter* sp., *Pseudomonas* sp., and *Azospirillium* sp. (El-Khawas and Adachi 1999). Due to its important role in plants, the level as well as distribution of IAA in plant tissue and endophytic production of IAA has gained a great deal of attention (Matsuda et al. 2005).

In addition to the IAA production, plant growth promoting bacteria (PGPB) are also shown to exhibit other properties like ACC deaminase, phosphate solubilization, siderophore production, etc. The enzyme ACC deaminase catalyzes degradation of 1-aminocyclopropane-1-carboxylic acid (ACC), the immediate precursor of ethylene, into α-ketobutyrate and ammonia and this inturn reduce the inhibitory effects of elevated level of ethylene. Plant associated bacteria can also have the capability to solubilise non-available phosphate to available form and there by enhance plant growth and yield (de Freitas et al. 1997). Siderophores are iron-chelating agents secreted by some microorganisms under iron-limiting conditions. Siderophore productions by some microorganisms make them successful in surviving several adverse environments and also make the iron limiting to plant pathogens (Miethke and Marahiel 2007).

Thus isolation and characterization of endophytes with diverse properties from unexplored sources will have much applications to manipulate plant growth promotion (Patten and Glick 2002; Sergeeva et al. 2007). In order to explore the promising potential of endophytes, diverse communities of endophytes should be isolated from various tissues of taxonomically diverse and metabolically distinct plants. Plants of Zingiberaceae, especially ginger are well known for the presence of structurally diverse bioactive metabolites including those of the gingerol group (Ramirez-Ahumada Mdel et al. 2006). Also ginger forms a model plant of the family where much interesting and unexplored rhizome specific metabolism is present. In addition to the complex chemical constituents, rhizome is also well known for its ability to survive under adverse conditions (Ramirez-Ahumada Mdel et al. 2006). So many interesting groups of microorganisms with diverse roles in plant physiology can be expected from the rhizome. However the growth promoting properties of endophytic bacteria from ginger has not yet been well studied. So studies on isolation and characterization of endophytic bacteria from ginger is very significant. In the current study four endophytic bacteria were isolated from ginger rhizome and one among the isolates was found to have the ability to produce IAA, ACC deaminase and siderophore.

## Materials and methods

### Isolation and characterization of endophytic bacteria

Rhizome of ginger (*Zingiber officinale)* was collected from Navajyothisree Karunakara Guru Research Centre for Ayurveda and Siddha, Uzhavoor, Kottayam and was used as the source material for the isolation of endophytic bacteria. The rhizome pieces of *Z. officinale* were washed with tap water to remove soil and were made to 1–2 cm long pieces. This was further treated with Tween 80 for 10 min with vigorous shaking. This was followed by wash with distilled water for several times to remove Tween 80. After the treatment with Tween 80, the samples were dipped in 70 % ethanol for 1 min and then treated with 1 % sodium hypochlorite for 10 min. The samples were then washed several times with sterilized distilled water and the final wash was spread plated onto nutrient agar plate (g/L; peptone 5, beef extract 2, yeast extract 3, sodium chloride 5 and agar 18, pH 7.0) as control. For the isolation of endophytic bacteria, the outer surface of the sterilized plant material was trimmed, the pieces were further macerated in Phosphate buffer saline (PBS) (g/L—sodium chloride 8, potassium chloride 0.2, disodium hydrogen phosphate 1.44 and potassium dihydrogen phosphate 0.24, pH 7.4) and was serially diluted up to 10^−3^ dilution. From this, 0.l mL was plated onto nutrient agar plates. All plates including the control were incubated at room temperature for 5 days and observed periodically for bacterial growth. Those batches of experiments where the bacterial growth, if any present, in the control plate were completely discarded. Morphologically distinct colonies as identified by colony characters were selected, purified and used for further studies.

### Identification of the isolates by 16S rDNA sequencing

Genomic DNA was isolated from all the bacterial isolates and was used as template for PCR. Primers used for the amplification of part of 16S rDNA were 16SF (5′-AgA gTT TgA TCM Tgg CTC-3′) and 16SR (5′-AAg gAg gTg WTC CAR CC-3′) and were selected based on the previous reports of Chun and Goodfellow (1995). PCR was carried out in a 50 μL reaction volume containing 50 ng of genomic DNA, 20 pmol of each primer, 1.25 units of Taq DNA polymerase (Bangalore Genei), 200 μM of each dNTPs and 1X PCR buffer. PCR was carried out for 35 cycles in a Mycycler™ (Bio-Rad, USA) with the initial denaturation at 94 °C for 3 min, cyclic denaturation at 94 °C for 30 s, annealing at 58 °C for 30 s and extension at 72 °C for 2 min with a final extension of 7 min at 72 °C. The PCR product was checked by agarose gel electrophoresis, purified and was further subjected to sequencing. The sequence data was checked by BLAST analysis (Zhang et al. 2000). The phylogenetic analysis of the 16SrDNA sequences of the isolates obtained in the study was conducted with MEGA 5 using neighbor-joining method with 1,000 bootstrap replicates (Tamura et al. 2011).

### Screening of isolates for plant growth promoting properties (PGP)

#### IAA production

The bacterial isolates were inoculated into 20 mL of nutrient broth supplemented with 0.2 % (v/v) of l-tryptophan and incubated for 10 days at 28 °C. After incubation, the culture was centrifuged at 3,000 rpm for 20 min and the supernatant was used for analysing indole 3 acetic acid production (Rahman et al. 2010). Initially one mL supernatant was mixed with 2 mL of Salkowski reagent and tubes were incubated in dark for 30 min. The development of the red color was observed as the indication for positive result. Uninoculated growth medium was used as negative control. The IAA positive isolates were further inoculated into 200 mL of nutrient broth supplemented with 0.2 % (v/v) of l-tryptophan and incubated for 10 days at 28 °C. After incubation the cell free extract was collected by centrifugation at 3,000 rpm for 20 min. The supernatant was then acidified to pH 2.5–3.0 with 1 N HCl and was extracted twice with ethyl acetate. The extracted ethyl acetate fraction was vacuum dried in a rotary evaporator at 40 °C. The dried powder was dissolved in 1 mL of methanol (MeOH) and stored at −20 °C. For confirmation of presence of IAA, the methanol extract of culture supernatant was subjected to reverse-phase HPLC analysis on a Supelcosil LC-18 column with a flow rate of 1 mL min^−1^ as described by Jensen et al. (1995). Elution was performed with mixture of H_2_O and MeOH (60:40), both containing 0.5 % acetic acid. Elution was monitored at 280 nm by shimadzu UV–Vis Detector model SPD 10A.

#### ACC deaminase production

The ACC deaminase production of the endophytic bacterial isolates from ginger were screened using the methods described by Jasim et al. (2013). For this, the isolates were inoculated on to DF salts minimal medium (potassium dihydrogen phosphate 4 g/L, disodium hydrogen phosphate 6 g/L, magnesium sulfate heptahydrate 0.2 g/L, ferrous sulfate heptahydrate 0.1 g/L, boric acid 10 μg/L, manganese(II) sulfate 10 μg/L, zinc sulphate 70 μg/L, copper(II) sulfate 50 μg/L, molybdenum (VI) oxide 10 μg/L, glucose 2 g/L, gluconic acid 2 g/L, citric acid 2 g/L, agar 12 g/L) amended with 0.2 % ammonium sulphate (w/v). The bacterial growth in this media after 2 days of incubation was considered as positive result.

#### Phosphate solubilization

The endophytic bacterial isolates were screened for phosphate solubilization using the procedure described by Jasim et al. (2013). For this, Pikovskaya medium (g/L—glucose 10, tri-calcium phosphate 5, ammonium sulphate 0.5, sodium chloride 0.2, magnesium sulphate heptahydrate 0.1, potassium chloride 0.2, ferrous sulfate heptahydrate 0.002, yeast extract 0.5, manganese (II) sulfate dehydrate 0.002, agar 20, pH 7.0) containing 2.4 mg/mL bromophenol blue was used. The media inoculated with the isolates were incubated for 48 h and was observed for the formation yellow zone around the colony due to the utilization of tricalcium phosphate present in the medium.

#### Siderophore production

The isolates were checked for the production of siderophores on blue agar CAS medium containing chrome azurol S (CAS) and hexadecyltrimethylammonium bromide (HDTMA) as indicators (Schwyn and Neilands 1987). The blue agar CAS medium was prepared by adding 850 mL of autoclaved MM9 salt medium [added with 32.24 g piperazine-N, N′-bis 2- ethanesulfonic acid (PIPES) at pH 6], 100 mL of blue dye, 30 mL of filter sterilized 10 % Casaminoacid solution and 10 mL of 20 % glucose solution. The blue agar medium was aseptically poured on to sterile plates and allowed to solidify. All the bacterial isolates obtained were inoculated into the CAS medium and incubated at 28 °C for 24 h. Development of yellowish orange halo around the colonies was taken as the indication for the production of siderophore.

## Results and Discussion

Fresh and cleaned ginger rhizomes were used for the isolation of endophytic bacteria. The rhizomes were surface sterilized to remove the epiphytic microorganisms. The surface sterilization procedure for the isolation of endophytic bacteria as standardized in the experiment was quite satisfactory as no growth appeared on the control plate. Also, adequate number of colonies obtained in the nutrient agar plates which were inoculated with plant samples macerated in PBS. Based on the distinct colony characteristics, the bacterial isolates obtained were grouped into four and were named as ZoB1–ZoB4. As no microbial growth was observed in control plate, the isolates ZoB1–ZoB4 obtained in the study can be considered as endophytic bacteria of ginger.

Molecular identification of the isolates was done by sequencing part of the 16S rDNA. The amplification of the 16S rDNA was confirmed by agarose gel electrophoresis. The PCR product was gel eluted and sequenced. The 16S rDNA sequences of the bacterial isolates were submitted to NCBI under the accession numbers as explained in Table 1.

**Table 1:** 16S rDNA sequence analysis of endophytic bacterial isolates from *Zingiber officinale*

Plant material	Endophytic isolate	NCBI accession number	Sequence analysis	
			Closest NCBI database match with accession number	Percentage of identity
Ginger rhizome	ZoB1	JN835212	*Bacillus barbaricus* JF727665	99
Ginger rhizome	ZoB2	JN835214	*Pseudomonas putida* JN596120	99
Ginger rhizome	ZoB3	JN835215	*Stenotrophomonas maltophilia* FN645734	99
Ginger rhizome	ZoB4	JN835216	*Staphylococcus pasteuri* JF510533	98

The sequence data of the 16S rDNA was subjected to BLAST analysis. As 16S rDNA gene sequence provide accurate grouping of organism even at subspecies level it is considered as a powerful tool for the rapid identification of bacterial species (Jill and Clarridge 2004). The sequence analysis of 16S rDNA sequences of ZoB1, ZoB2, ZoB3 and ZoB4 showed its maximum identity of 99 % to *Bacillus barbaricus* (JF727665), 99 % to *Pseudomonas putida* (JN596120), 99 % to *Stenotrophomonas maltophilia* (FN645734) and 98 % to *Staphylococcus pasteuri* (JF510533), respectively. Therefore, the isolates ZoB1–ZoB4 can be considered as strains of *Bacillus* sp*., Pseudomonas* sp*., Stenotrophomonas* sp, and *Staphylococcus* sp, respectively (Table 1). The presence of these organisms as endophytes has not been reported from ginger rhizome.

**Fig. 1:**
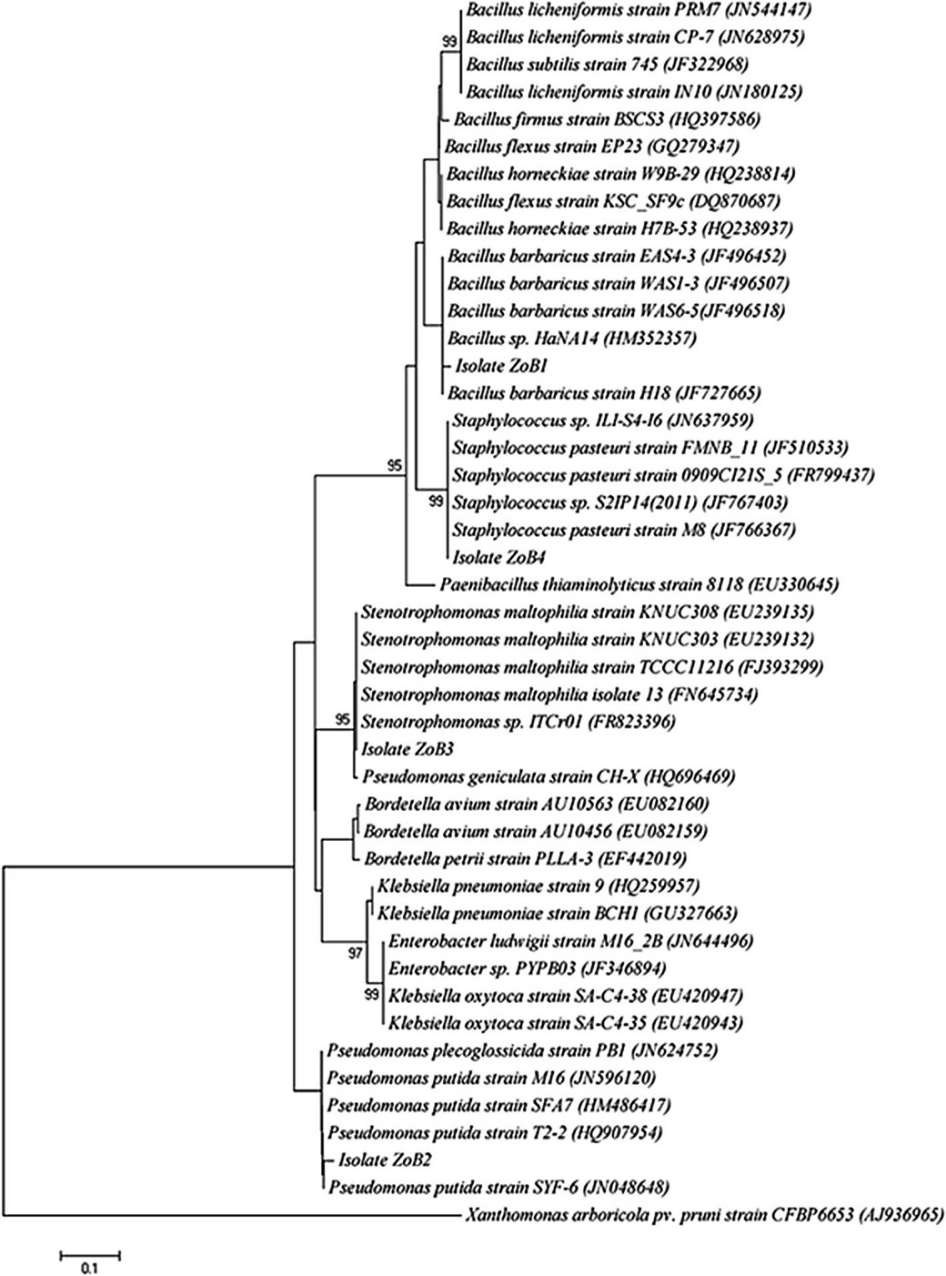
Phylogenetic analysis of 16S rDNA sequences of the bacterial isolates (ZoB1–ZoB4) from ginger along with the sequences from NCBI. The analysis was conducted with MEGA5 using neighbor-joining method

Strains of *P. putida* and *S. maltophilia* were previously reported as endophytes from other plants like *Populus* sp. (Taghavi et al. 2009). *S. pasteuri* strains were previously identified as endophyte from plants like Arabidopsis and Soyabean (Panchal and Ingle 2011). From the comparative analysis, it is very clear that bacteria like *P. putida*, *S. maltophilia*, and *S. pasteuri* are present as endophytes in wide variety of plants. However, *Bacillus barbaricus* was reported as an endo-lithosphere associated bacteria of *Musa* sp. (Thomas and Soly 2009). As information on the presence of *Bacillusbarbaricus* as an endophyte is very limited, the presence of related strain as endophyte in ginger rhizome may be taken as an indication of its specific or limited association as an endophyte. Thus, it can be considered that endophytic bacteria present in ginger rhizome, include those species with wide range of host specificity and those with limited distribution. However, the presence these organisms in specific habitat of ginger make them much more interesting. This is because these stains can expect to have strain specific plant growth promoting potential. The phylogenetic analysis of 16S rDNA sequence of the isolates along with the sequences retrieved from the NCBI was carried out with MEGA 5 using the neighbor-joining method with 1,000 bootstrap replicates. The result of phylogenetic analysis showed distinct clustering of the isolates (Fig. 1).

**Fig. 2:**
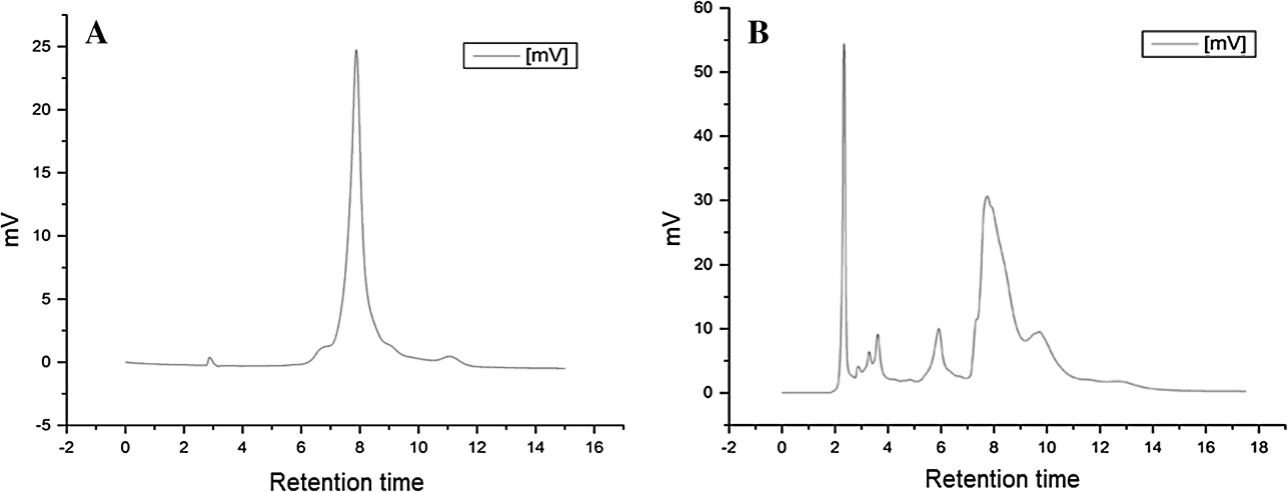
HPLC analysis carried out using water and methanol, both containing 0.5 % acetic acid in the ratio 60:40 on reversed phase C18 column with a flow rate 1 mL min^−1^ and detected under UV at 280 nm. **a** Positive control (indole 3 acetic acid only), **b** methanolic extract from ZoB2 (Pseudomonas sp.)

The culture supernatant of the bacterial isolates ZoB1–ZoB4 were checked for indole 3 acetic acid production calorimetrically. For this, the supernatant of the culture was treated with Salkowski reagent as explained by Rahman et al. (2010). Among the four endophytic isolates, ZoB2 gave positive result for the production of indole 3 acetic acid. The positive reaction was confirmed by comparing this with positive control, which had pure indole 3 acetic acid and a negative control which had uninoculated culture medium. The positive result appeared had a color similar to that of the positive control. Thus, ZoB2 (*Pseudomonas* sp.) was found to have the ability to produce IAA (Table 2). The production of the indole 3 acetic acid was also confirmed by HPLC. For this, the extracts were run on a C18-reversed phase column and absorbance was measured at 280 nm. The pure indole 3 acetic acid produced a peak at 8 min retention time and the crude extract had a predominant peak at the same retention time (Fig. 2). This confirmed the production of indole 3 acetic acid by the bacterial isolate ZoB2 (*Pseudomonas* sp.) from ginger. Even though ZoB1 also showed positive result when treated with Salkowski reagent, the results could not be confirmed by HPLC.

**Table 2:** Growth promotion capability of the isolated endophytic bacterial isolates

Name of the Isolates	Isolate Identified as	Plant Growth promoting properties			
		Phosphate solubilization	ACC Deaminase	Siderophore production	IAA production
ZoB1	*Bacillus sp.*	−	−	**+**	−
ZoB2	*Pseudomonas sp.*	−	**+**	**+**	**+**
ZoB3	*Stenotrophomonas sp.*	−	−	**+**	−
ZoB4	*Staphylococcus sp.*	−	−	−	−

Some endophytic microorganisms have the potential to synthesize IAA. This may be a reason for the increased growth promotion of some plants when the plant is colonized with endophytes (Shi et al. 2009). For the microbial synthesis of IAA in tryptophan-dependent route, tryptophan is used as the precursor. There are different pathways that can lead to tryptophan-dependent microbial production of IAA. The various pathways for IAA biosynthesis include tryptophol, tryptamine, indole-3-pyruvic acid and indole-3-acetamide pathways (Gravel et al. 2007). The ability of the production of IAA different species of *Pseudomonas* was reported by many authors (Karnwal 2009). Even there are reports that suggest the ability of *P. putida* GR12-2, when inoculated on seeds can result in 2–3 fold increase in the length of seedling roots (Glick et al. 1986; Caron et al. 1995).

Even though the presence of *Pseudomonas* sp. as endophyte and its ability to produce IAA has already been reported from other plants including both monocot and dicots, its reports from ginger is limited. This makes the present finding much more interesting. The strain of *Pseudomonas* sp. identified in this study can have important growth regulating role in ginger rhizome. The strain may also have unique features to survive in the unique chemical environment of rhizome under various conditions. Confirmation of this by further experiments may pave the way for exploring the potential application of the isolate in ginger yield enhancement.

All the four isolates (ZoB1-ZoB4) were screened for the production of ACC deaminase on DF salts minimal medium amended with 0.2 % ammonium sulphate. ZoB2 (*Pseudomonas sp.*) was found to be positive for ACC deaminase production as indicated by its growth in the media (Table 2). Glick et al. (2007) suggests that some microbes can utilize the ACC as nitrogen source from the exudates of roots or seeds. This decrease in the levels of ACC and ethylene may prevent the ethylene-mediated plant growth inhibition. Endophytic microbes with these capabilities residing inside the host plants can benefit the host by reducing the stress and increasing the plant growth (Hardoim et al. 2008). Alizadeh et al. (2012) has explained the application of the ACC deaminase which has been synthesised by different genera of *Pseudomonas* in increasing the senescence of the plants. The endophytic bacterial isolates were also screened for phosphate solubilization, but the results were negative for all of the isolates.

The endophytic bacterial isolates were screened for siderophore production using the chrome azurol S (CAS) agar. Among the four endophytic isolates, ZoB1 (*Bacillus sp.*), ZoB2 (*Pseudomonas sp.*) and ZoB3 (*Stenotrophomonas sp.*) were found to have the ability to produce siderophore (Table 2). The formation of orange halo around the colonies due to the chelation of iron was the indication for production of siderophore. The formation of orange halo is as a result of the production of siderophore, which removes the iron from the dye complex that changes the color of the medium from blue to orange (Schwyn and Neilands 1987). Siderophores producing bacteria can sequestrate the limited iron and thereby reduce its availability for growth of phytopathogens. Thus, they enable the plant growth promotion indirectly (Alexander and Zeeberi 1991). Different species of *Bacillus* have been reported to have the ability to produce of siderophores even that of petrobactin type (Gardner et al. 2004; Wilson et al. 2010). Many reports reveals the ability of both gram negative bacterial isolates (*Pseudomonas* sp.) and bacterial genera of *Bacillus* and *Rhodococcus* that belongs to the gram-positive group with the capability to produce siderophores (Tian et al. 2009). Structural studies suggest that there are more than 50 structurally related siderophores like pyoverdins, that are produced by different species of *Pseudomonas* (Abdallah 1991; Budzikiewicz 1993).There are various reports that suggest the ability of *Stenotrophomonas maltophilia* to synthesize different types of siderophores using the universal CAS assay method. Chhibber et al. (2008) reported the production of ornibactin type siderophore by *Stenotrophomonas maltophilia* and Ryan et al. (2009) mentioned its ability to produce the catechol type siderophore compound enterobactin based on their recently sequenced genomes.

## Conclusion

The results from the study demonstrated the diverse community of endophytic bacteria associated with ginger rhizome. Among these endophytic bacterial isolates obtained, *Pseudomonas* sp. (ZoB2) isolated from the rhizome was found to have the ability to form Indole 3 acetic acid as confirmed by HPLC analysis. The isolate was also found to have the capability to produce ACC deaminase and siderophore which have high impact on growth of the plant. Hence, this isolate can be considered to have growth promoting effect in ginger.
